# Clinical observations in acute neurology: A historical explanation of the present

**DOI:** 10.1111/ene.16241

**Published:** 2024-02-25

**Authors:** Eelco F. M. Wijdicks

**Affiliations:** ^1^ Neurosciences Intensive Care Unit Mayo Clinic Hospital Saint Marys Rochester Minnesota USA

**Keywords:** animal research, clinical signs, coma, discovery, history

## Abstract

**Background:**

The history of the development of acute neurologic disease as a biologic mechanism is of interest. Equally important is how it translated to the bedside and how the clinical examination differentiated itself.

**Methods:**

This paper reviews primary sources pertaining to acute neurologic conditions described mostly in the 19th and 20th century. A review of monographs, treatises, textbooks, and peer‐reviewed articles was conducted.

**Results:**

The evolution of clinical signs and syndromes associated with dynamic intracranial pathologies was predicated on the idea that animal studies informed clinicians, who then linked clinical signs to these observations. A dominant theme is that innovative technologies could trace acute processes through all their various stages, affording a complete picture of the disease process. Just as clinical descriptors of central nervous system processes evolved, the presentation of acute neuromuscular respiratory failure became better defined. Once practices incorporated these acute clinical signs, textbooks cemented their “gold standard” status with relative impunity.

**Conclusions:**

The practise of acute neurology and neurocritical care must find out what, historically, others were seeking but could not find. Patterns of clinical presentation in acute neurology are sufficiently recognizable to guide practise decisions. Although, the currently well‐documented clinical syndromes of acute neurologic conditions (at presentation and during deterioration) have been taught for generations, practitioners have noted that they lack consistency and predictability. History also taught us that part of this improved knowledge came with designated units―a clear example of how protean systems (not always innovative neurologists or neuroscientists or technologies) shaped the history of neurology.

## INTRODUCTION

History in neurology leads us to discoveries, surprises, and insights in clinical observations. The body of knowledge of acute neurology was marginal (at least compared to more chronic conditions). Clinical observations were derived from animal experiments and neuropathology. Anatomists and physiologists worked on multiple aspects of the respiratory system, pulmonary function, and circulation associated with acute neurologic disease. Often, as happened with brain herniation, more detailed observation changed conventions.

This article captures the evolution of clinical signs and syndromes and dynamic intracranial pathologies. Subsequently, breakthroughs occurred when hospital units for patients with similar pathologies markedly improved recognition of disease, deterioration, and outcomes.

## BROAD THEMES OF THE HISTORY OF ACUTE NEUROLOGY

Readers of medical history encounter a constant struggle with language barriers, poor translations, apocryphal stories, personal sketches, and obscure writing, sprinkled with untruths, leading to a skeptical interpretation of events. However, these problems are less applicable to the contemporary history of medicine, and the history of acute neurology is recent enough to be trustworthy. Nevertheless, difficulties with provenance remain.

Acute neurology differs from the neurology of chronic conditions, but both have existed since time immemorial. Early physicians were confronted with paralysis, convulsions, coma, and head injury. Prognosis―namely, distinguishing fatal from nonfatal―was their skill. Management (now known as “neurocritical care”) was not typically a goal until the 1960s or later. (For example, Plum and Posner's celebrated *The Diagnosis of Stupor and Coma* [1] [1966] largely focused on diagnosis, just as the title implied).

Historians accept that modern technologies fuelled human progress; this concept also applied to medicine. Breakthroughs in neurologic diagnosis and care depended on breakthroughs in physics; most notably, the sensational, transformative development of the computed tomography (CT) scan, which essentially changed our approach to patients [2]. One of the first published CT scans (by neuroradiologist James Ambrose) showed an intracranial hemorrhage, an acute but previously unseen neurological condition [3]. Reluctance of neurologists and neurosurgeons delayed reliance on the technology, but once overcome, recognition of acute conditions became more accurate. Widespread availability of CT scanning in the late 1970s enabled rapid identification of the cause of virtually everything inside the brain. While undoubtedly helpful for neurologists, this represented a significant advance for neurosurgeons and eliminated the need for emergency cerebral angiograms and “blind” exploratory surgery (burr holes) to detect acute subdural hematomas. Subsequently, magnetic resonance imaging displayed damaged and displaced brain structures even more accurately. These technologies could trace acute processes through their various stages, affording a complete picture. They also changed the understanding of clinical signs and syndromes, eliminating interpretations through inference.

Finally, no new specialty starts with a big bang or seminal event and many neurologists from prior centuries can claim an imprint on the development of clinical signs and management of the sickest patient.

## CLINICAL PATTERNS OF ACUTE MASS EFFECT IN THE BRAIN

**FIGURE 1:**
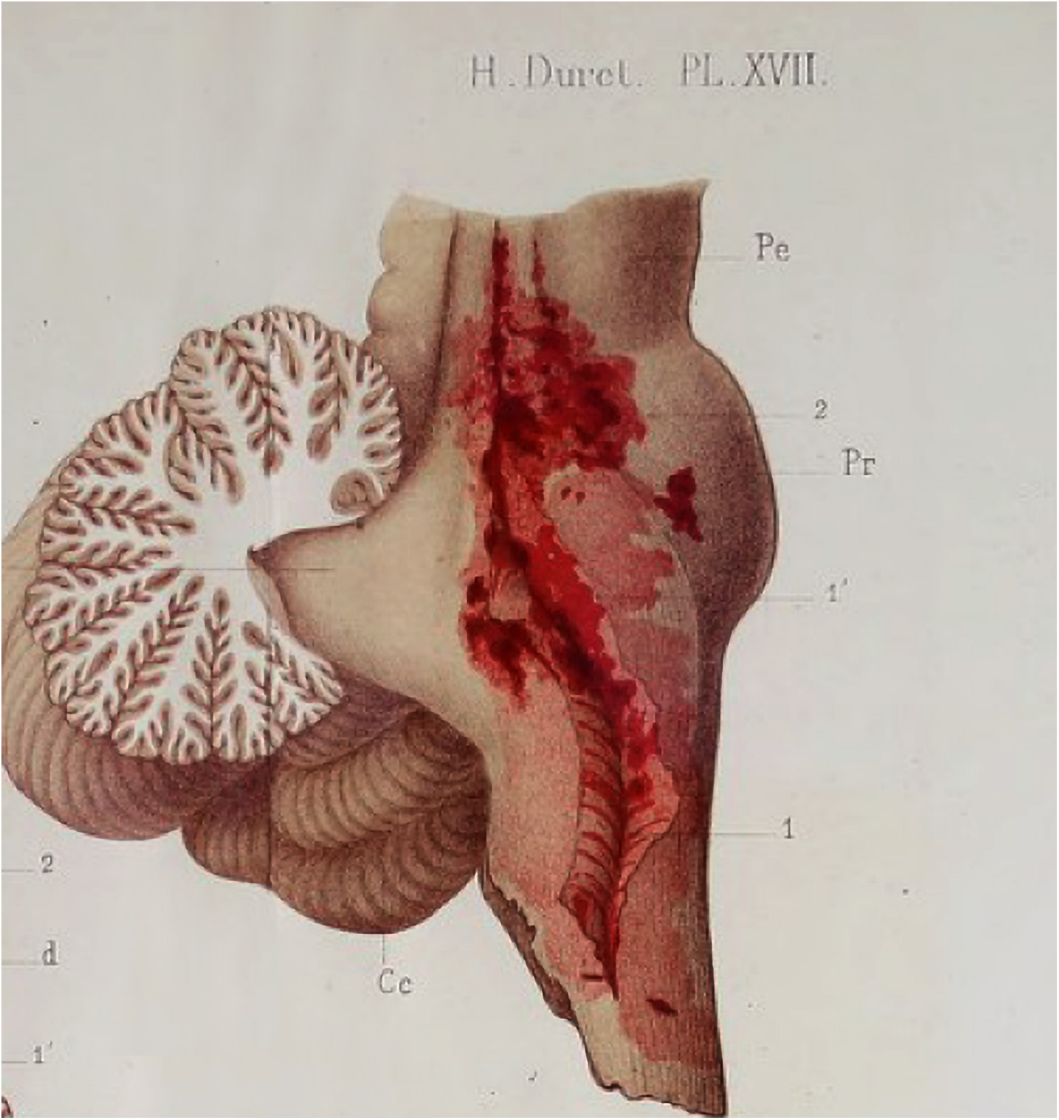
Pontine and medulla hemorrhage associated with intracranial pressure surge [5]. From Duret, *Etudes Expérimentales sur les Traumatismes Cérébraux. Thèse pour le Doctorat en Médicine. Etude expérimentales sur les traumatismes cérébraux. Versailles imprimerie et stéréotypie Cerf et Fils*; 1878 [5].

Study of acute brain‐mass dynamics involves two important, novel observations—brain shift worsens consciousness and unaddressed shift leads to permanent brainstem injury. Supratentorial tissue displacement damages infratentorial tissue structures. Leon Rostan, a 19th‐century physician at the Salpêtrière Hospital, made one of the earliest clinicopathologic correlations between decline in consciousness and later deterioration in patients with cerebral softening. Noting brain compression at autopsy, he recalled that these patients displayed pupil dilation, which then became fixed, and their respiration became stertor [4]. Shifting brain tissue clearly injured the brainstem. Many rapidly developing intracranial mass lesions correlated with pontomedullary hemorrhages. In 1878, Duret found lesions in 30% of traumatic and spontaneous cerebral hemorrhages (Figure 1) [5]. He described the clinical and pathological features of *les phénomènes de choc* after injection of water or gelatine into the cranium [5] but also noted clinical signs, including sudden acceleration of blood pressure, respiratory arrest, slow pulse, and *‘tétanisme’* (posturing), as well as pathology. This observation occurred decades before Cushing's triad. Duret hypothesized that shock waves in the cerebrospinal fluid expanded in the aqueduct of Sylvius, causing hemorrhage under the fourth ventricle. (This explanation, based on Bernoulli's law, derives from hydrostatic pressure being strongest in the immediate post‐stenotic area).

The previous century witnessed impressive transformations in understanding the mechanisms of coma, the role of increased intracranial pressure, patterns of brain tissue shift, and clinical correlates. But our understanding of brain herniation remained incomplete. Some explanations were one‐dimensional, focused on pathology, and extrapolated from slow‐growing brain tumors. Nonetheless, clinicians accepted the familiar refrains of “herniation” such as fixed pupils, posturing, agonal breathing patterns, and rostrocaudal deterioration. Some signs resonate because they offer an explanation where no definitive one exists. Admittedly, no recent comprehensive study has unravelled the enigma of how and when brain tissue shift damages the thalamus and upper brainstem, and observing the comatose patient at the bedside shows that clinical deterioration rarely follows characteristic patterns.

C. M. Fisher's detailed paper, ‘Neurologic Examination of the Comatose Patient,’ [6] revealed remarkable understanding of the variable presentation of impaired consciousness. Fisher emphasized ocular signs in a comatose patient as “the most important of the entire examination”. After seeing 10 cases with clinical‐pathological correlation, Fisher described ocular bobbing (‘an “intermittent down and up” conjugate movement of the eyes’), which he correlated with pontine pathology. Other novel ocular signs included “wrong‐way eyes”, pontine miosis, ocular agitation, doll's eyes, eye closure and blinking, and reflex blepharospasm. Famed for his diligence, Fisher highlighted two findings regarding the depth of coma: eyelid tone and the length of time the eyes remain open after the examiner opens them. New motor observations included bilateral decerebrate posturing resulting from acute lesions involving the supratentorial motor system. Other observations include ventral midbrain syndrome (decerebrate posture with full extraocular movements and fixed pupils in tentorial brainstem compression), ptosis with mass supratentorial lesions, relative silence of cerebellar pressure cone, the 1½ syndrome, and adventitious movements.

Fisher also questioned the extrapolation of animal studies to humans. These included Sherrington's cat experiments, the concept of uncal herniation (midbrain involvement rather than herniating tissue compressing the oculomotor nerve), and even tendon reflexes in coma (“not of great value in assessing the state of the nervous system” [7]).

Earlier, McNealy and Plum's published observations of clinical deterioration and elucidation of two brain‐herniation syndromes [8] and offered the first description of the clinical course of a deteriorating patient with expanding mass effect. The clinical patterns of rostrocaudal deterioration, which correlated to structures involved and mass location, provided generations of neurologists with a framework to pursue at the bedside. Displacements were grouped into either central or uncal syndrome. Central syndrome comprised diencephalon, mesencephalon, and pontine involvement successively, and the uncal syndrome, due to its encroaching on the oculomotor nerve, started with a fixed, dilated pupil followed by mesencephalon and pons involvement in a similar caudal direction. McNealy and Plum emphasized changes in pupils, eye movements and position, worsening motor responses to abnormal posturing (decerebrate or decorticate), and changes in respiratory patterns (Cheyne‐Stokes, central hyperventilation, and ataxic and apneustic breathing). Despite being based on a small number of closely observed patients, it became the last word on the subject.

Another revelation came with the discovered loss of all brainstem reflexes, apnea, and progressive hypotension in a patient with a rapidly deteriorating brain and no explanation other than a catastrophic lesion; this became known as cerebral death or brain death (the vacuous term “death by neurologic criteria” is staging a comeback). The 1950s invention of the positive‐pressure mechanical ventilator led to recognition of progressive loss of brainstem function, breathing drive, and vascular tone, observations not previously apparent because patients succumbed from failure to protect the upper airway. It also became apparent that injury of the lower pons and medulla oblongata of mechanically ventilated patients inevitably led to irrevocable loss of brainstem reflexes. The normal dying sequence was a rostrocaudal deterioration of brainstem function; lost mesencephalic and pontine reflexes preceded functional loss of respiratory and pressure centers in the medulla causing apnea and hypotension. This led to a definition of death by brainstem criteria despite increasingly sophisticated systems able to maintain the other bodily systems. Fully disconnected from brain signals, the body became dependent on intrinsic pacemakers. This simple neurologic observation launched a reexamination of the crucial role of the brainstem in human vital functions and a new insight (i.e., our mind and thinking parts of the brain subjugated by the brainstem).

Is the rest history? A detailed review of the discovery of clinical signs may tell us otherwise.

## REFINING AND REVISING THE CLINICAL EXAMINATION

During the 18th and 19th centuries, clinical examination of the brainstem was fragmented. Textbooks emphasized disease and presented the examination of cranial nerves only when it related to a disease process. Georg Monrad‐Krohn developed the first systematic examination of the cranial nerves but omitted examination during coma [9].

Experimental studies revealed that pupils dilate and become fixed to light in acute brain lesions. In the early 1800s, German internists and surgeons Von Leyden, Naunyn, and Bergmann noted in their experimental studies that pupils dilate with increasing intracranial pressure. They considered pupillary changes were a result of medulla oblongata ischemia because their appearance so closely correlated to hypertension and periodic breathing.

Pupillary changes have fascinated clinicians, and their significance outweighs any other sign in coma. The earliest clues to this time‐honored sign date back to 1867, when Sir Jonathan Hutchinson observed unilateral pupillary dilatation in a patient but dismissed its significance [10]. Nearly two decades later, Sir William Macewen recognized its import in a treatise on the pupil “…the patient was generally insensible at the onset when both pupils were dilated and fixed. As the patient recovered consciousness, one pupil became normal while the other remained dilated and fixed; this being on the side of the lesion.” [11]

**FIGURE 2:**
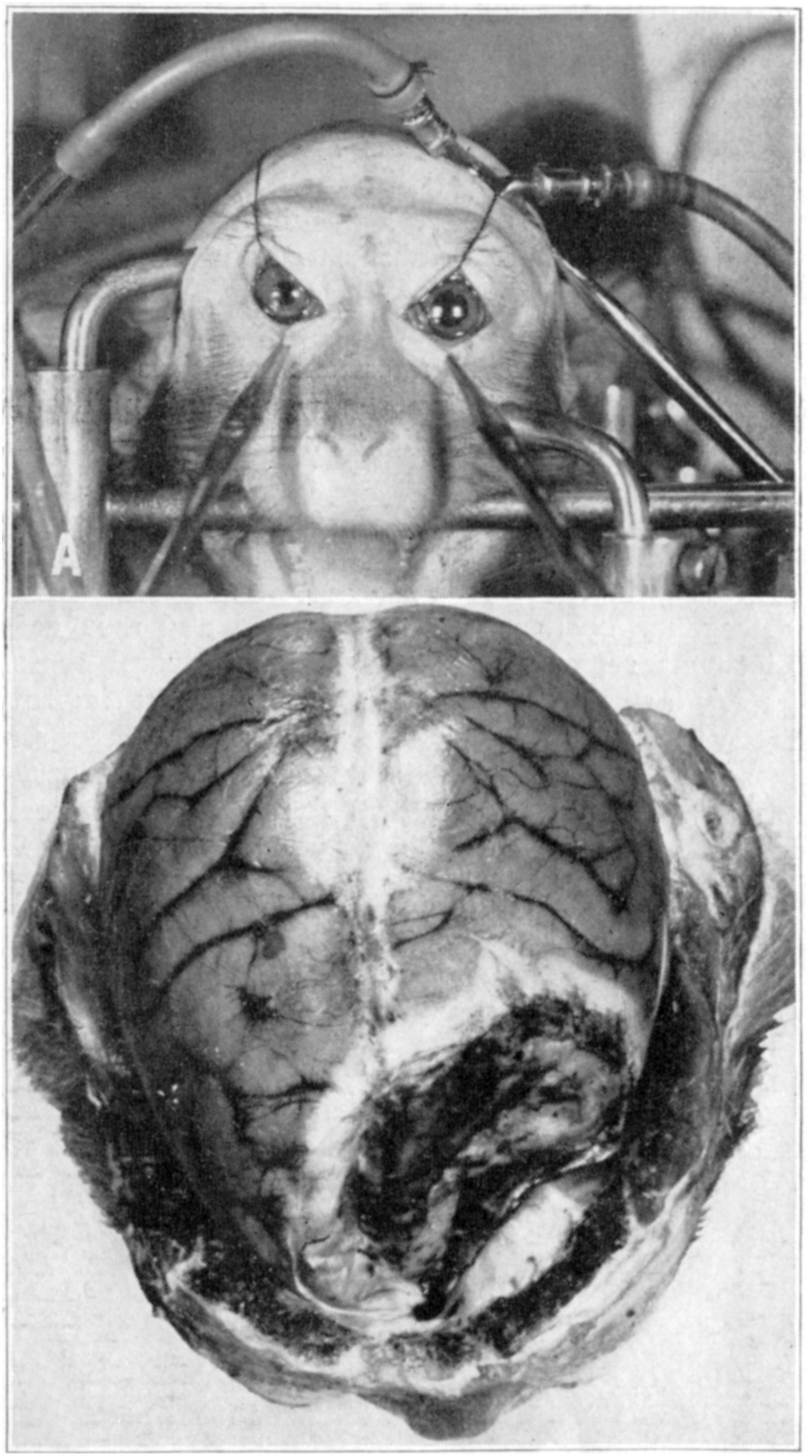
Demonstration of fixed and dilated pupil, from Reid and Cone [14]. Used with permission of the American Medical Association.

German surgeon Bergmann believed that a fixed, dilated pupil indicated a lesion in the cortex. In his classic work *Deutsche Chirurgie: Die Lehre von den Kopfverletzungen*, he located oculomotor dysfunction in the frontal lobe. He also described widening of the pupil ipsilateral to the lesion [12, 13]. In their classic studies, Reid and Cone clearly confirmed the correlation between fixed dilatation of the pupil and herniation by a mass (Figure 2) [14], and Jennett and Stern [15] replicated the experiment in cats. Much later, Ropper suggested acute angulation of the third nerve over the clivus due to brainstem displacement in an autopsy study [16]. The explanation of the pupil mechanism is not definitive; more than one mechanism may be operative. How the opposite pupil enlarges with transtentorial herniation remains a mystery. Bilateral central (at the nucleus level) third‐nerve damage seems a more likely mechanism.

The corneal reflex historical origin is unknown, but the reflex arc is placed in a narrow territory in the pons. A much larger tested territory is associated with the vestibulo‐ocular reflexes with a circuit from the third nerve to the seventh‐nerve nucleus, thus involving a broad brainstem region (high midbrain‐low pons). G. H. Klingon made the initial discovery of the impaired vestibulo‐ocular reflex in coma; he demonstrated conjugate‐movement disorders of the eyes after cold‐water stimulation [17]. Disconjugate ocular responses (abduction of the eye with the opposite eye frozen) correlated in a comatose patient with demyelination in the tegmentum. In 1957, Nathanson et al. [18] described the possible usefulness of oculocephalic and caloric responses in comatose patients, including patients with completely absent oculocephalic reflex and caloric stimulation, when treated with barbiturates. Massive brainstem lesions from basilar artery occlusion or swollen glioblastoma with brainstem hematoma illustrated the clinicopathological correlation. These patients had disconjugate ocular movements; however, the presence or absence of corneal reflexes and pupillary light reaction were uncorrelated with findings on oculocephalic and caloric tests. Patients with a tonic ocular deviation regained consciousness, while patients missing caloric stimulation and oculocephalic reflexes did not. Thus, oculocephalic and caloric tests indicated coma depth and, when absent, signaled a poor prognosis. Furthermore, Ethelberg and Vaernet demonstrated abnormal conjugate eye movements, such as internuclear ophthalmoplegia, in three cases of supratentorial space‐occupying lesions. These earlier studies pioneered the use of clinical tests to assess brainstem function [19].

Other classic clinical signs require scrutiny. First, clinicians noted extensor rigidity with head retraction in comatose patients, most notably with cerebral hemorrhage extending into the ventricles. (Sir Charles Sherrington, a Nobel laureate in medicine and physiology, described decerebrate rigidity in transected animal experiments, which others confirmed [20, 21]).

Walsche hypothesized that midbrain lesions, lesions of the forebrain or ventricular hemorrhages hindered activity of the midbrain centers [22]. He noted, “yet the lesion does not end here and in almost all of them, there is clear evidence of a progressive and ultimate fatal interference with the function of the vital medullary centers.” Decerebrate posturing, after traumatic brain injury, was an acknowledged sign of poor prognosis [23]. Its localizing value in humans is less understood; it is observed in midbrain lesions or lesions involving injury to both hemispheres without brainstem injury or displacement. Moreover, worsening to flaccidity or change to withdrawal or decorticate responses does not clearly correlate with outcome. Both decorticate (pathologic flexion) and decerebrate (pathologic extension) responses indicate severe structural injury. Many patients have a decorticate response on one side and a decerebrate response on the other, or even alternating in the acute stage. Often the “worst” response correlates with the hemispheric lesion. The responses must be different manifestations of a similar lesion rather than being precisely localisable.

## THE WEAKENING MUSCLES OF RESPIRATION

Since the early 1800s, biologists recognize enlargement of the thoracic cavity in all directions during inspiration. The lungs expand to fill the created space. When muscles relax, gravitational force and elastic reaction of the thoracic wall and pulmonary tissue restore the thorax to its original size; inspiration requires muscle contraction, but expiration is passive.

In the late 1800s, physiologist Henry Newell Martin refocused on respiratory muscles, attempting to elucidate the additional role of the internal intercostal muscles and their function as accessories during diaphragm failure and extreme dyspnea [24]. These muscles have a secondary inspiratory function, and over time, investigators also found an inspiratory function for the sternocleidomastoid muscles, which activate during labored breathing. Shaefer's *Textbook of Physiology* in 1900 clearly identified them without specific attribution but also mentioned abdominal muscles for forced expiration [25]. Duchenne's (largely forgotten) treatise on lung‐muscle mechanics [26] is most notable. He is better known for describing muscular dystrophy. Duchenne concluded that diaphragm paralysis was not necessarily fatal. He may have been the first to recognize paradoxical breathing (expansion of the chest and inward movement of the abdomen with inspiration) in patients with diaphragm paralysis. These basic respiratory biomechanics remain the foundation of our understanding.

Over several decades, US centers have provided care for respiratory distress, recognizing that oropharyngeal failure signals insufficiency of respiratory muscles. Already in 1945, a reported 60 cases from Massachusetts General Hospital highlighted respiratory failure and, specifically, oropharyngeal failure [27]. Later, Rowland et al. reported 39 fatal cases between 1930 and 1955 [28]. They urged physicians to watch for warning signs, use sedatives cautiously, initiate early mechanical ventilation, and control for infection.

Campbell and Bramwell in *Brain* [29] alerted UK (and US) physicians to myasthenia gravis—and its potential for critical worsening. They offered detailed descriptions of 60 patients, 23 with fatal outcomes. Frequent involvement of bulbar muscles in their cases and ptosis, with accompanying weakness of the neck muscles, impaired the compensatory mechanism of “throwing back the head”. The paper emphasized choking, nasal fluid regurgitation, diminished palatal reflex, lowered voice tone suggesting weakness of the vocal cord adductors, and an inability to thrust out the cheek and maintain protrusion of the tongue. Measured circumferentially, the difference in chest movements between inspiration and expiration did not exceed a quarter inch. Dyspnea with exertion was common and there were “unaccountable attacks of breathlessness, during which the patient is in danger”. Accumulated mouth sputum accompanied dyspneic attacks, leaving patients unable to swallow or spit: “The tongue appears to sink back into the mouth”. Retracting the tongue markedly improved symptoms. The fatal cases resulted from choking, “dyspneic attacks”, or “dyspneic attacks with pneumonia”. (Autopsies found nothing anatomically wrong in the medulla oblongata.) Rowland emphasized that “involvement of respiratory muscles with consequent attacks of dyspnea is a symptom of gravest significance” and warned of death occurring during an attack. The detail in these early communications is striking.

Another major acute neuromuscular disease, acute inflammatory demyelinating polyneuropathy, was originally described by George Guillain, Jean Barré, and André Strohl. It later became known as Guillain–Barré syndrome (GBS; for unknown reasons, the eponym excluded Strohl, likely because he merely performed the electrodiagnostic studies [30].) Protective of his discovery, Guillain opined that Landry's case of acute ascending paralysis (with worse outcome and respiratory failure) [31] was a different condition and said including Landry's name confused the nomenclature (*Une confusion nosographique absolue*). Apart from the unexplained fever, Landry's descriptions strongly resemble the disease in severely affected patients as we currently know it.

Gradually, the literature reported more severe cases of GBS. Until the 1950s, patients could die from respiratory arrest. A 1951 record from Massachusetts General Hospital described a 13‐year‐old female GBS patient with “tired” breathing and “pharyngeal mucus”, who died from fatal aspiration pneumonia on the sixth hospital day. The “respirator”’ in this report was probably a tank ventilator [32].

**FIGURE 3:**
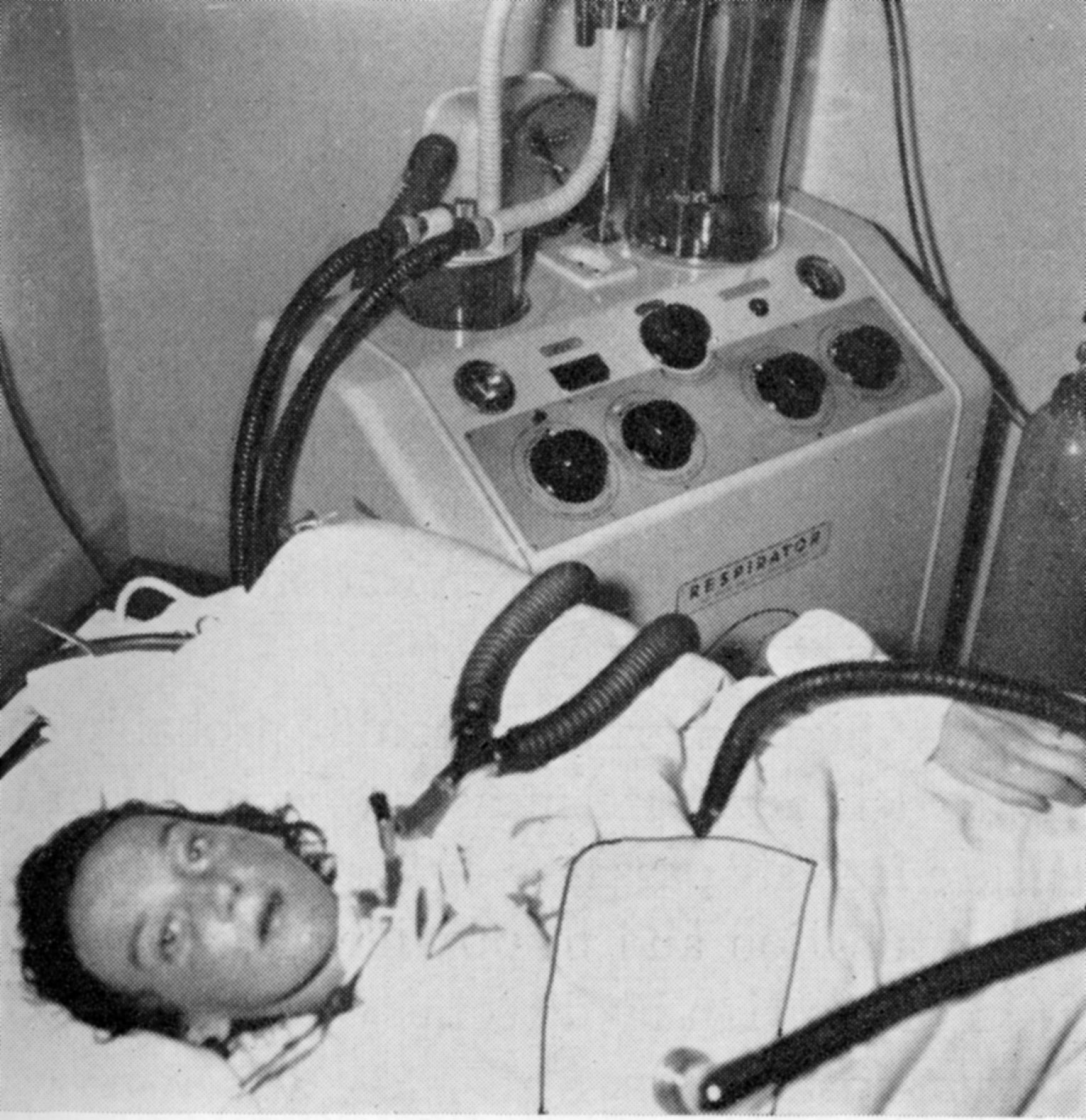
Guillain–Barre syndrome with positive pressure ventilation and tracheostomy, from Bendz [33]. Used with permission of the American Medical Association.

Swedish physician Per Bendz's article on GBS respiratory care presented three patients placed in a cuirass who “died with gurgling mucus in the pharynx”. [33] The fourth case, a rapidly progressing 28‐year‐old woman, with dysphagia and respiratory distress leading to cyanosis and absent diaphragmatic breathing, survived due to a prompt tracheostomy and connection to an Engström respirator (Figure 3) [33]. This patient, successfully weaned, is likely among the first published cases of successful respiratory care and positive‐pressure mechanical ventilation in severe GBS.

Russell, Baker, and Plum's report of airway and respiratory recognition and management suggestions was a major contribution [34]. It is commonly misreported that neurologists entered this arena after anesthesia and other specialities defined critical care medicine. Detailed guidance to help general neurologists recognize acute respiratory failure was paused until neurocritical care emerged as a specialty [35, 36]. Even now, the recent GBS guideline provides insufficient clinical information beyond the modified Erasmus GBS Respiratory Insufficiency Score (mEGRIS) and unvalidated tests for lung mechanics [37] (and mEGRIS has no clinical assessment of respiratory function [38]). Clinical evaluation of neuromuscular respiratory failure requires no additional tests and is recognized by bedside observation: inhalation after each sentence, hoarseness, weak cough, difficulty clearing secretions and drinking sips of water, using sternocleidomastoid muscles with each breath, and paradoxical breathing. All these symptoms ofen lead to reduced SpO2 on oximeter.

## THE PRESENT PLACE OF ACUTE NEUROLOGY

History points to major watershed moments in acute neurology and care of the neurocritically ill patient. First, after major hospitals acquired CT scanners, we learned that cerebral hemorrhage is not static, traumatic contusions and subdural hematoma appear suddenly, and acute hydrocephalus in aneurysmal subarachnoid hemorrhage requires immediate ventriculostomy. Moreover, patients with ominous clinical and CT signs and a basilar artery occlusion dramatically improve if the clot can be physically removed. Cerebral metabolism and intracranial pressure measurements had far less impact and, for many of us, remain controversial as potential modifiers of outcome. (The BEST TRIP trial on intracranial pressure treatment in traumatic brain injury, which found no difference in outcome, compared treatment protocols without assessing the value of knowing intracranial pressure [39].).

Second, the clinical syndromes of acute neurologic conditions (at presentation and when deteriorating) are now well documented and consistently taught. Neurointensivists know reasonably well what to expect. They know how a patient with subarachnoid hemorrhage can look after 7 days; how a mechanically ventilated patient with GBS recovers and with what trajectory; how traumatic, disastrous‐appearing brain injuries may still resolve satisfactorily in young individuals (It defines the specialty much better than knowing which mode of ventilation).

Looking back at other developments, we must ask how new knowledge influenced decisions, especially concerns about the appropriateness of major neurosurgical procedures, and documentation of success versus failure. Neurosurgeons could randomly decide whether certain preoperative presentations warranted emergency surgery. Development of extensor posturing was traditionally associated with futility. Decerebrate posturing had prognostic import, especially when associated with supratentorial space‐taking lesions (i.e., hemorrhage, abscess, or tumor) [40, 41]. However, these dicta changed, and except with loss of brainstem reflexes, neurosurgical intervention often proceeds in patients with a supratentorial mass. Other clinical conditions, such as cerebellar swelling, brainstem compression, or acute hydrocephalus, when treated immediately, can restore pontomesencephalic brainstem reflexes. Although studies in the preoperative prediction of neurologic examination remain scarce, motor responses, pupil reflexes and size, and eye position became less critical in neurosurgical decision making when better‐than‐expected recoveries resulted. Patients with acute ventricular enlargement, fixed pupils, and extensor posturing *can* improve rapidly after ventriculostomy placement. Patients with large acute cerebellar lesions pressing on the brainstem *can awaken* after lesion evacuation. Since the 1960s, clinical signs of coma were used to predict awakening and outcome. It remains a formidable assignment but must be done regularly. Neurologists understandably struggle.

**FIGURE 4:**
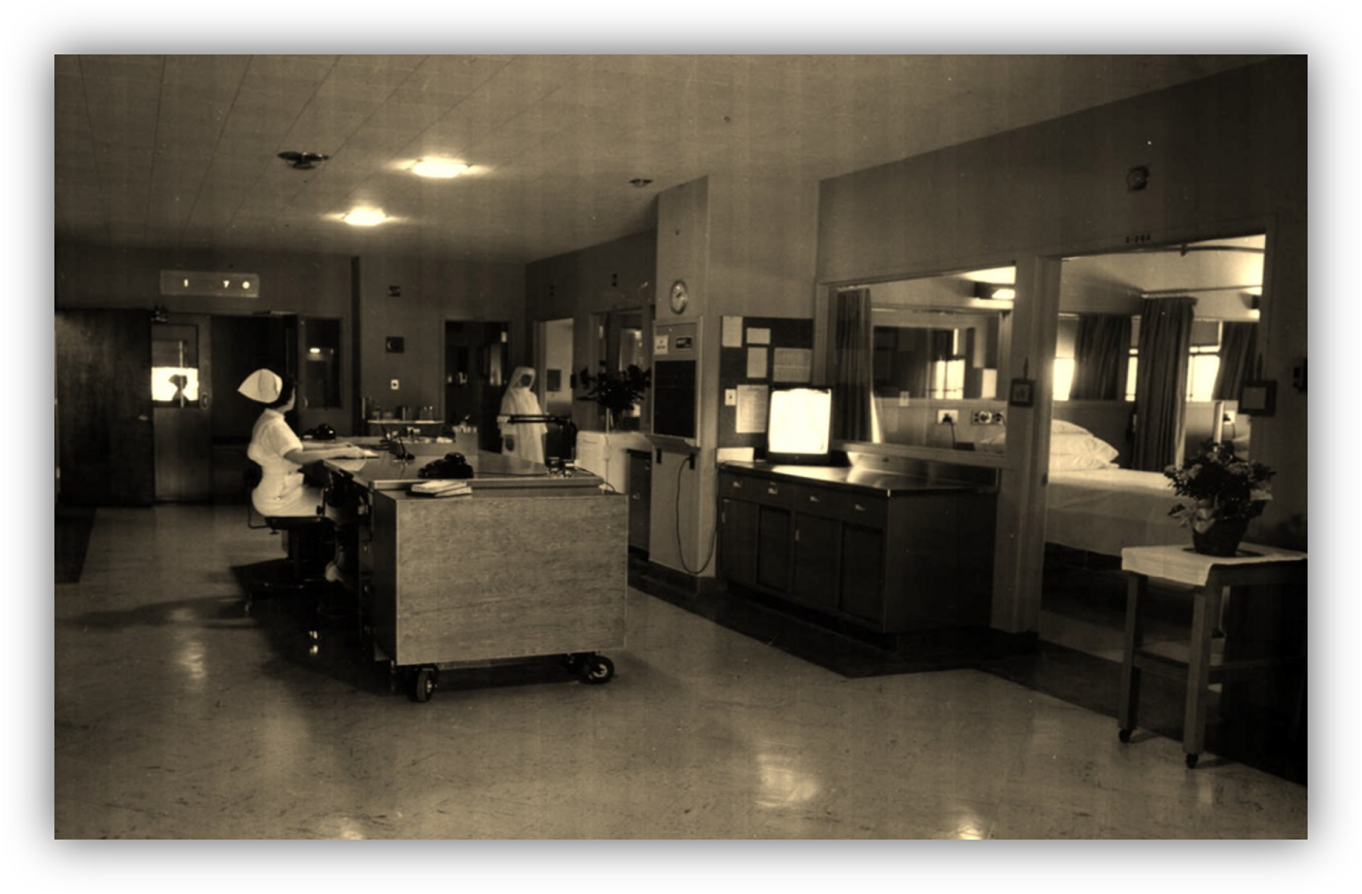
Early neurosciences intensive care unit. A 12‐bed unit divided into six double rooms with glass partitions between the rooms and a centralized desk that would allow an overview of all patients, from Wijdicks et al. [42], used with permission of the *Mayo Clinic Proceedings*.

Specialized “units” proliferated in Europe, the United Kingdom, the United States, and elsewhere, each representing different expertise. Surgical recovery units became surgical intensive care units (ICUs); patients with treatable cardiac arrhythmias after myocardial infarction or requiring complex hemodynamic support led to coronary care units. Patients with multi‐organ trauma ended up in trauma ICUs, coordinated by trauma surgeons and, later, surgical intensivists. Similarly, hospitals opened burns units. Neurosurgeon Walter Dandy opened one of the first neuroscience ICUs at Johns Hopkins University in 1932, by refurbishing a ward to admit his sickest postoperative neurosurgical patients. Twenty years later, the Mayo Clinic opened the first newly built, combined neurosciences ICU at Saint Mary's Hospital and admitted neurologic and neurosurgical patients (Figure 4) [42]. Pandemics, particularly the 1950s polio pandemics, promoted specialized respiratory units in Europe, the United Kingdom, and the United States.

Improved knowledge of acute neurologic conditions grew out of these designated units of patients with specific neurologic disorders in a single space. Attending healthcare professionals developed strategies to improve care, a clear example of how systems, rather than technologies, shaped the history of neurology. Units dedicated to stroke patients decreased mortality, limited morbidity, and allowed more patients to return home with retained independence. Recognizing deterioration with acute brain injury, neurologic complications of critical illness, and recognition and triage in acute neurology emergency rooms in the 1990s were important landmarks. Neurologists learned in the units and published their experiences. Moreover, acceptance of a diverse subspecialty that includes other non‐neurologic disciplines created cross‐pollination of knowledge and further opportunities to improve complex care of these conditions.

The 1990s witnessed the first‐time publication of textbooks and publications that discussed causes of deterioration in acute brain injury and treatment and prognostication of coma [43, 44, 45]. Important prognostic and brain death determination guidelines followed.

We can easily envision a patient‐and‐computer interface providing detailed, sophisticated, online, artificial intelligence‐modulated brain functioning information after a major injury. The next decades will reveal how much remains out of reach. Can data recorded by invasive or noninvasive monitoring replace neurointensivists' clinical assessments? Opinions vary between grandstanding and serious doubt [44, 45]. Others have identified so‐called endotypes in traumatic brain injury using statistical models or machine learning [46] but with clinical examination truncated to a summed Glasgow Coma Score and many metabolic derangements. These endotypes might foretell trajectories. In these models, laboratory abnormalities receive more attention than granularity of the neurologic examination.

We all prefer clinical observation and detailed examination. Is the neurology of neurocritical care such a crucial part of pathophysiology that it cannot be compromised, or worse, replaced by devices or tests? Do pupillometers measuring new anisocoria designate a new CT scan? Unlike critical care medicine, where corrections of laboratory derangements are front and center, acute clinical neurology and neurocritical care remain in the discovery stage.

Clinical research is innate in acute neurology because of its immediate translation to patient benefit. Uncertainties are plentiful. Urgent clinical questions are: (1) How do brain displacement, shift, and herniation relate to clinical changes? (2) When is neurosurgery futile? (3) Do more granular neurologic examinations improve recognition of trajectories and prognostication? (4) How do we best observe and record patients with acute neuromuscular respiratory disease, and what bedside signs betray respiratory peril? and (5) How are patients best weaned from mechanical ventilators? Research in this field requires common domains and collaborative efforts.

A rapidly deteriorating patient strikes fear into the hearts of many physicians. Neurointensivists and hospitalists know that no patient follows a preset pattern, and contradictions occur. Our protean history also shows that uncritically accepting clinical patterns stifles rethinking of mechanisms. The practice of acute neurology and neurocritical care must continue to explore what others historically sought without success. Once we understand these clinical trajectories, we can simulate them and improve proficiency [47, 48].

## AUTHOR CONTRIBUTIONS

Eelco F.M. Wijdicks: conceptualization; data curation, writing and editing drafts.

## CONFLICT OF INTEREST STATEMENT

There are no conflicts of interest.

## Data Availability

Data sharing is not applicable to this article as no new data were created or analyzed in this study.
